# ZnFe_2_O_4_/GQDs Nanoparticles as Peroxidase Mimics for Sensitive and Selective Colorimetric Detection of Glucose in Real Samples

**DOI:** 10.3390/mi16050520

**Published:** 2025-04-28

**Authors:** Claudia Cirillo, Mariagrazia Iuliano, Maria Sarno

**Affiliations:** 1 Department of Physics “E.R. Caianiello”, University of Salerno, Via Giovanni Paolo II, 132, 84084 Salerno, Italy; 2 Centre NANO_MATES, University of Salerno, Via Giovanni Paolo II, 132, 84084 Salerno, Italy

**Keywords:** glucose detection, ZnFe_2_O_4_/GQDs nanocomposite, enzyme mimic, peroxidase-like activity, low detection limit (LOD)

## Abstract

Glucose detection is critical in addressing health and medical issues related to irregular blood levels. Colorimetry, a simple, cost-effective, and visually straightforward method, is often employed. Traditional enzymatic detection methods face drawbacks such as high costs, limited stability, and operational challenges. To overcome these, enzyme mimics or artificial nano-enzymes based on inorganic nanomaterials have garnered attention, but their cost and susceptibility to inactivation limit applications. This study presents a ZnFe_2_O_4_/GQDs nanocomposite as an innovative enzyme mimic, addressing key requirements like low cost, high stability, biocompatibility, and wide operational range. Synthesized using a simple and inexpensive method, the composite benefits from the synergistic interaction between ZnFe_2_O_4_ nanoparticles and graphene quantum dots (GQDs), resulting in excellent magnetic properties, high surface area, and functional versatility. The material demonstrated remarkable sensitivity with a detection limit of 7.0 μM across a range of 5–500 μM and achieved efficient peroxidase-like activity with Km values of 0.072 and 0.068 mM and Vmax of 4.58 × 10⁻^8^ and 8.29 × 10⁻^8^ M/s for TMB and H_2_O_2_, respectively. The nanocomposite also exhibited robust recyclability, retaining performance over six reuse cycles.

## 1. Introduction

**Scheme 1:**
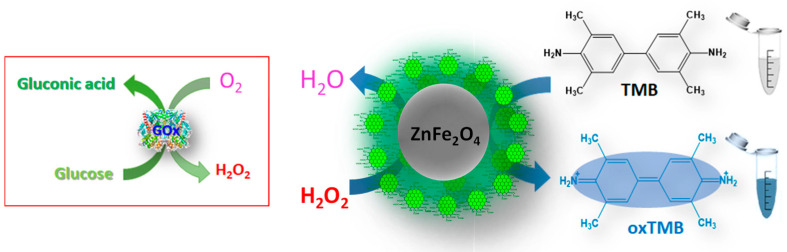
Schematic diagram of glucose determination by ZnFe_2_O_4_/GQDs NPs.

Glucose is an essential molecule in living organisms. It is found in numerous industrial products and serves as a key source of energy [1,2,3,4]. According to fasting tests, normal human blood glucose levels fall within a narrow range of approximately 3 to 8 mM [5,6]. Irregular blood glucose levels have long been an alarming problem in the field of health and medicine because they pose a formidable threat that affects people’s quality of life and makes them suffer from many dangerous diseases. Diabetes is a metabolic condition characterized by elevated glucose concentrations, which causes chronic harm and breakdown of multiple organs such as the heart, kidneys, eyes, and blood vessels. According to statistics from the World Health Organization (WHO), the number of diabetes cases increased by 314 million between 1981 and 2014. In 2019, an estimated 1.5 million people died directly from diabetes. For this reason, detecting glucose has become a topic of considerable interest and research effort. Currently, glucose detection is of great interest due to its several advantages, ranging from medical applications to ecological implications. Several methods for glucose detection—electrochemical [7], chromatographic [8], fluorometric [9], and colorimetric [10]—have been developed. Each approach has its strengths and weaknesses. In contrast to alternative methods, colorimetry offers simple operation, affordability, rapidity, practicality, and effective visual detection [11,12]. The colorimetric assessment of glucose involves an indirect analytical technique. When the glucose oxidase (GOx) enzyme is present, glucose undergoes oxidation, generating hydrogen peroxide. The latter, when coupled with another enzyme like horseradish peroxidase (HRP), oxidizes 3,3,5,5-tetramethylbenzidine (TMB) into a blue ox-TMB compound. Consequently, the glucose level can be indirectly quantified by measuring the absorbance of the blue solution. Nonetheless, enzymes have inherent limitations, including high costs associated with separation and purification, narrow operational parameters, and reduced stability in varying environmental conditions, which significantly hinder their practical applications, particularly in catalytic reactions [13]. Recently, there has been considerable interest in enzyme mimics or artificial nano-enzymes based on inorganic nanomaterials, which offer enhanced stability and activity even under severe reaction conditions [14,15]. Artificial nano-enzymes exhibit distinct advantages such as efficiency, simplicity, stability, and superior catalytic performance compared to natural enzymes. Inorganic nanomaterials have been extensively investigated using various strategies to mimic the structures and functions of natural enzymes, including metal-based nano-enzymes (e.g., Fe, Ag, Au, Ti, and Pt) [16]; carbon-based nanostructures (e.g., graphene oxide, carbon nanotubes, and fullerene); and other classes of nanomaterials [17]. However, the practical applications of noble metals are restricted due to their high cost and susceptibility to deactivation. Conversely, economically viable metal oxide-based nano-enzymes (such as CeO_2_, ZnO, CuO, NiO, VO_2_, Co_3_O_4_, and Fe_3_O_4_) [18,19], exhibiting diverse morphologies, have garnered increasing attention for their comparable catalytic activity. In this context, among various binary magnetic metal oxides, zinc ferrite (ZnFe_2_O_4_) has garnered significant attention due to its exceptional properties, including superior magnetic behavior, excellent dispersibility, high chemical stability, efficient catalytic performance, ease of separation, and low toxicity of zinc ions [20,21,22]. Moreover, ZnFe_2_O_4_ nanoparticles exhibit inherent peroxidase-like catalytic activity and have been successfully utilized for the colorimetric detection of organic molecules [19,23]. However, the limited efficiency in separating electrons and holes within bare ZnFe_2_O_4_ nanoparticles results in moderate catalytic activity. To tackle this challenge, an effective approach involves further functionalizing ZnFe_2_O_4_ nanoparticles to harness the synergistic effects among different components. Currently, carbon-based nanomaterials have demonstrated tremendous potential in numerous fields. Among them, carbon dots (CDs) represent a relatively novel type of carbon nanomaterial. Their fundamental structure comprises both sp^2^- and sp^3^-hybridized carbon atoms, featuring a plethora of functional groups [24]. Extensive studies have underscored CDs’ promise across traditional and emerging domains, encompassing photoluminescence (PL), photoelectrochemical sensing, catalysis, imaging, and biomedical applications, highlighting their superior characteristics compared to other carbon allotropes [25,26]. Nevertheless, certain potential applications of CDs remain largely unexplored. Specifically, leveraging CDs as catalysts and supports in catalytic reactions, driven by their surface chemistry, adjustable surface area, and electronic properties [27,28], is still at an early stage [24]. Notably, crystalline and sp^2^-hybridized graphene quantum dots (GQDs), composed of single, double, and multiple layers of graphene sheets with lateral dimensions under 100 nanometers and diameters ranging from 3 to 20 nanometers [29,30], are anticipated to differ from amorphous and sp^3^-hybridized carbon quantum dots (CQDs) [31]. GQDs exhibit a myriad of properties, including stable photoluminescence, robust stability, chemical inertness, strong fluorescent activity, high biocompatibility, low toxicity, eco-friendliness, cost-effectiveness, and the ability for surface functionalization [32]. Due to the quantum confinement effect and conjugated edge effects, the band gap can be adjusted, allowing them to conjugate with more delocalized molecular orbitals. Their superior properties of high electron mobility, e.g., increase in conductivity with decreasing sp^2^/sp^3^ ratio [33], high specific surface area, and abundance of surface functional groups (hydroxyl, carboxyl, ether groups, etc.) enhancing stability and water solubility [34], promote their applications [35,36,37,38,39,40,41] even more in catalysis [31,42]. Recently, graphene-based materials and their derivatives have surfaced as enzyme mimetics owing to their benefits over natural enzymes, including cost-effectiveness, straightforward preparation, high stability, and the potential for long-term storage. Studies have demonstrated that graphene quantum dots (GQDs) exhibit stronger peroxidase-like activity than graphene oxide (GO) and carbon nanotubes (CNTs), attributed to their small size and high density of active sites [43], surpassing that of carbon quantum dots (CQDs) as well [44,45,46]. However, like metal-based nanomaterials, their application is hindered by challenges in separation [47]. In this work, a highly dispersible, simply recoverable, and reusable ZnFe_2_O_4_/GQDs NPs nanocomposite was designed to be synthesized through a simple synthetic route and inexpensive materials to ensure, at low costs and biocompatibility, high stability and activity in a wide range of operative conditions, e.g., pH, temperature, range of analytes, etc., thanks to high surface area for catalysis and tailored physical properties. It was synthesized for easy and efficient action in both steps of the indirect glucose detection. GQDs, enriched with a good proportion of functional groups, were synthesized by citric acid pyrolysis [48]. They were adopted as supports during the synthesis of ZnFe_2_O_4_ nanoparticles to produce ZnFe_2_O_4_/GQDs NPs, enjoying synergistic interaction between GQDs and ZnFe_2_O_4_ NPs and endowed with well-preserved magnetic behavior. In particular, GQDs, consisting of a single-atom-thick graphene sheet rich in oxygen-containing functional groups, conjugate the nanoparticle’s periphery. They result in a light and very thin quasi-continuous layer, preserving nanoparticle structure and magnetic properties, favoring dispersion stability and avoiding aggregation, to amplify peroxidase-like activity. The as-prepared ZnFe_2_O_4_/GQDs nanoparticles were employed to mimic the oxidation of TMB and used for the indirect detection of glucose (see Scheme 1). The first step involved the detection of H_2_O_2_, which can be readily extended to figure out the concentration of glucose, considering that H_2_O_2_ evolved in the catalytic reaction of glucose with glucose oxidase (GOx). This study also aimed to develop a sensor that was stable in a wide range of conditions and even at very low concentrations and capable of being operational in “real” environments, i.e., directly on blood samples. Recyclability was evaluated by reusing the nanomaterials several times. Finally, the ZnFe_2_O_4_/GQDs were applied for the quantitative detection of glucose in the serum samples.

## 2. Materials and Methods

### 2.1. Materials

Citric acid, FeCl_3_·6H_2_O, and ZnCl_2_ were purchased from Sigma-Aldrich (St. Louis, MO, USA). All chemicals used in these experiments were of analytical grade and were used as received, without any further purification. Deionized water was used for all experimental procedures.

### 2.2. Synthesis of GQDs

GQDs were synthesized through direct pyrolysis of citric acid [49]. Initially, 2.0 g of citric acid was placed in a 250 mL three-necked flask and heated to 200 °C using a heating mantle, without the use of an inert atmosphere, until the citric acid melted completely. The reaction proceeded until the pale yellow liquid turned orange–red, indicating the formation of GQDs, which typically took about 10 min. The hot molten product was immediately added dropwise over 10 min to 40 mL of NaOH solution (20 mg/mL prepared in distilled water) under continuous mechanical stirring. Finally, the pH of the resulting GQDs solution was adjusted to 7 using NaOH. The solution was then stored at 4 °C.

### 2.3. Synthesis of ZnFe_2_O_4_/GQDs NPs

The ZnFe_2_O_4_@GQDs nanocomposite was synthesized using a modified Massart method [50]. Initially, 4 mmol of FeCl_3_·6H_2_O was added to 50 mL of GQDs solution at room temperature under a nitrogen atmosphere with vigorous stirring. The temperature was then raised to 80 °C, and 2 mmol of ZnCl_2_ was slowly added to the solution containing Fe^3+^/GQDs while maintaining vigorous stirring. Subsequently, a 25 wt.% ammonia solution was gradually added dropwise over 5 min to adjust the pH to 10, facilitating the formation of ZnFe_2_O_4_@GQDs. The mixture was stirred for 30 min under these conditions to ensure uniform precipitation. After the reaction, the black precipitate was collected using an external magnetic field, washed several times with deionized water and ethanol, and dried at room temperature.

To ensure that enzymatic activity did not result from leaching of metal ions into the buffer, the nanocomposite was incubated in a 60 mmol/L acetate buffer solution for two days. Following magnetic separation to remove the ZnFe_2_O_4_@GQDs, no metals were detected in the supernatant, indicating effective protection by the GQDs against metal leaching.

### 2.4. Characterization Techniques

UV–visible spectroscopy of the GQDs solution was performed using a Thermo Scientific™ Evolution 60S UV–visible spectrophotometer (ThermoFischer Scientific, Waltham, MA, USA). Scanning electron microscopy (SEM) and transmission electron microscopy (TEM) analyses were carried out using a TESCAN-VEGA LMH (operated at 230 V, Brno, Czechia) and FEI Tecnai (operated at 200 kV, Hillsboro, OR, USA), respectively, to investigate particle size, morphology, and crystal structure. The Brunauer–Emmett–Teller (BET) method was used to determine the specific surface area (SSA) using a Costech Sorptometer 1042 (Costech Microanalytical OÜ, Tallinn, Estonia), following pretreatment at 150 °C for 60 min under helium flow. Thermogravimetric analysis was carried out using a TGA 2 Mettler Toledo (Columbus, OH, USA) instrument under airflow at a heating rate of 10 °C/min. FT-IR spectra were recorded using a Nicolet^TM^ iS50 FT-IR spectrometer (ThermoFischer Scientific, Waltham, MA, USA). X-Ray Diffraction (XRD) (Billerica, MA, USA) measurements were performed using a Bruker D2 Phaser diffractometer (Billerica, MA, USA) and nickel-filtered CuKα radiation (λ = 1.5418 Å).

### 2.5. The Peroxidase-like Catalytic Activity of ZnFe_2_O_4_@GQDs NPs

To investigate the peroxidase-like catalytic activity of ZnFe_2_O_4_/GQDs NPs, the oxidation of 3,3′,5,5′-tetramethylbenzidine (TMB) in the presence of H_2_O_2_ was evaluated, as depicted in Scheme 1 on the right. The absorbance of oxidized TMB at 652 nm was measured across a wide range (1–250µM) of H_2_O_2_ concentrations to enable low-level detection using a UV–vis spectrometer.

Initially, a solution consisting of 2.37 mL of 0.1 M HAc-NaAc buffer (pH 4.0), 0.24 mL of ZnFe_2_O_4_/GQDs NPs suspension (1.0 mg/mL, diluted with water), 0.03 mL of TMB (10 mM, diluted with ethanol), and 0.36 mL of H_2_O_2_ (5 mM, diluted with water) was prepared. The mixture was incubated at 45 °C for 20 min and then immediately separated using an external magnet. The UV–vis absorbance at 652 nm of the supernatant was recorded to quantify the presence of ox-TMB.

Concurrently, several experimental parameters were optimized, including pH (ranging from 2.0 to 10), incubation temperature (ranging from 30 °C to 60 °C), and catalyst concentration (in the range of 0.2 to 2 mg/mL). These optimizations aimed to enhance the catalytic efficiency of ZnFe_2_O_4_/GQDs NPs in the colorimetric detection of TMB oxidation products.

### 2.6. Steady-State Kinetic Analysis of ZnFe_2_O_4_/GQDs NPs

To further explore the catalytic activity of ZnFe_2_O_4_/GQDs NPs, the apparent steady-state kinetic parameters for the catalyzed reaction were determined by varying the concentrations of TMB and H_2_O_2_. The experiments were conducted using 80 µg/mL of ZnFe_2_O_4_/GQDs NPs in 0.1 M HAc–NaAc buffer (pH 4.0), with either 5.0 mM H_2_O_2_ and varying concentrations of TMB (0.1–1.0 mM) or 0.8 mM TMB and varying concentrations of H_2_O_2_ (0.1–1.0 mM). After incubating for 5 min, the reactions were quenched with cold water. The absorbance of oxidized TMB at 652 nm was then measured, and the concentration of oxidized TMB was estimated using the Beer–Lambert law (molar extinction coefficient, ε = 3.9 × 10^4^ M^−1^ cm^−1^) [51].

The data obtained from the spectrophotometric analysis were fitted in the Michaelis–Menten equation:

(1) $$V=\frac{V_{max}\left[S\right]}{k_{m}+\left[S\right]}$$

Michaelis–Menten constant (*K_m_*) was also calculated using the Lineweaver–Burk plots from the following equation:

(2) $$\frac{1}{V}=\frac{k_{m}}{V_{max}\left[S\right]}+\frac{1}{V_{max}}$$

where [*S*] is the substrate concentration, *K_m_* is the Michaelis–Menten constant, and *V* and *V_max_* are the initial and maximum reaction rates, respectively.

### 2.7. Active Species Capturing

For deeper insights into the catalytic mechanism of ZnFe_2_O_4_/GQDs NPs, isopropanol, D-histidine, and P-benzoquinone were employed as reactive oxygen species (ROS) scavengers to track singlet oxygen (^1^O_2_), hydroxyl radicals (·OH), and superoxide anions (O_2_•), respectively [52]. In each case, 100 μL of scavenger was added to a reaction system comprising 100 μL of TMB (5 mM), 100 μL of ZnFe_2_O_4_/GQDs NPs (1 mg/mL), 100 μL of H_2_O_2_ (10 mM), and 2700 μL of NaAc-HAc buffer solution (pH = 4). After incubation for 10 min, the absorbance at 652 nm was measured. Additionally, OH production was assessed using terephthalic acid (TA) as a sensor [53]. Various concentrations of ZnFe_2_O_4_/GQDs NPs, 10 mM H_2_O_2_, and 0.5 mM TA were combined in HAc-NaAc buffer solution (pH = 4) at 45 °C for 5 min, followed by recording the fluorescence intensity of the system with increasing concentrations of ZnFe_2_O_4_/GQDs NPs.

### 2.8. H_2_O_2_ and Glucose Colorimetric Detection

Colorimetric detection of H_2_O_2_ was performed as follows: in 2.37 mL of HAc-NaAc buffer solution at pH 4.0, 0.24 mL of 1 mg/mL ZnFe_2_O_4_/GQDs NPs solution, 0.03 mL of 10 mM TMB, and 0.36 mL of different concentrations (1–250µM) of H_2_O_2_ were mixed. After incubating in a water bath at 45 °C for 20 min, the resultant mixture was separated by an external magnet and used for UV–vis measurement. The quantitative detection of glucose was performed as follows: 0.5 mL of aqueous solutions of glucose at various concentrations and 0.13 mL of 1.0 mg/mL GOx were placed in a 5 mL reaction vial and incubated at 37 °C for 20 min. Then, 2 mL of HAc-NaAc buffer solution (pH 4.0), 0.24 mL of ZnFe_2_O_4_/GQDs suspension (1.0 mg/mL, water suspension), and 0.030 mL of TMB solution (10 mM) were sequentially added. The mixture was incubated at 45 °C for 20 min and then immediately separated by an external magnet. Subsequently, the UV–vis spectrum of the supernatants at 652 nm was measured.

To detect blood glucose, a 4 mL blood sample was collected from three volunteers (all participants signed informed consent forms). The blood samples were incubated at 37 °C for 10 min and then centrifuged at 12,000 rpm for 5 min. After that, the supernatant solution was diluted 10 times using PBS (0.1M, pH 7.0). The diluted serum was then used in place of the standard glucose solution, as stated above, to detect glucose concentration.

### 2.9. Reusability Test

The reusability of ZnFe_2_O_4_/GQDs nanoparticles in glucose detection was evaluated using a glucose concentration of 0.5 mM. After each catalytic reaction, the nanoparticles were magnetically separated, thoroughly washed three times with 10 mL of PBS each time, then dried at 60 °C for 1 h, and reintroduced into a fresh solution containing GOx, glucose, and TMB. The absorbance of the supernatant was measured to assess the catalytic activity. This process was repeated for seven cycles to evaluate the nanoparticles’ reusability.

## 3. Result and Discussion

### 3.1. Preparation of ZnFe_2_O_4_/GQDs Nanocomposites

**Figure 1:**
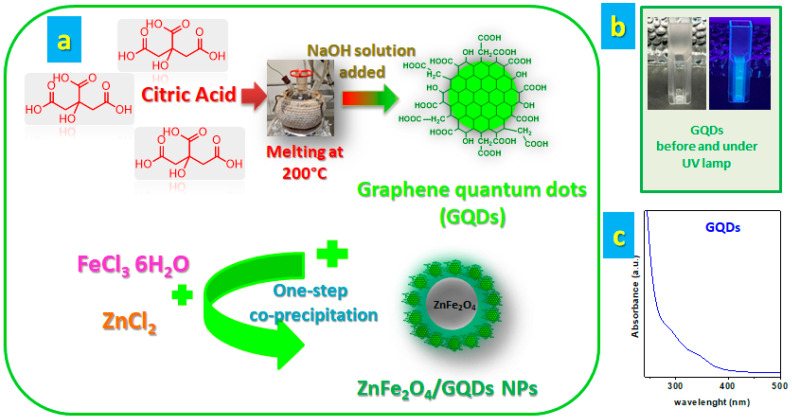
Schematic representation of GQDs and ZnFe_2_O_4_/GQDs NPs preparation (**a**); optical images of GQDs aqueous solution (DI water) excited at 365 nm (**b**); room temperature UV–visible absorption spectra of GQDs (**c**).

Two straightforward preparation steps for ZnFe_2_O_4_/GQDs nanocomposites were carried out, as depicted in Figure 1a. Initially, GQDs were synthesized via direct pyrolysis of citric acid, used as a carbon precursor. Heating to 200 °C induced the carbonization of citric acid, yielding GQDs. The resultant aqueous GQDs solution exhibits a light-yellow color under visible light and a light-blue color under 365 nm UV light, as shown in digital photographs in Figure 1b. The UV–vis absorption spectrum of GQDs is presented in Figure 1c, revealing distinct peaks at 290 nm and 340 nm. These peaks correspond to transitions attributed to π-π* and sp^2^ clusters within the structure [54]. GQDs consist of single-atom-thick graphene sheets featuring oxygen-containing functional groups on both planes and edges. These functional groups serve as anchoring and confinement sites, facilitating the in situ nucleation and growth of magnetic nanoparticles, as depicted in Figure 1a.

### 3.2. X-Ray Diffraction Studies

**Figure 2:**
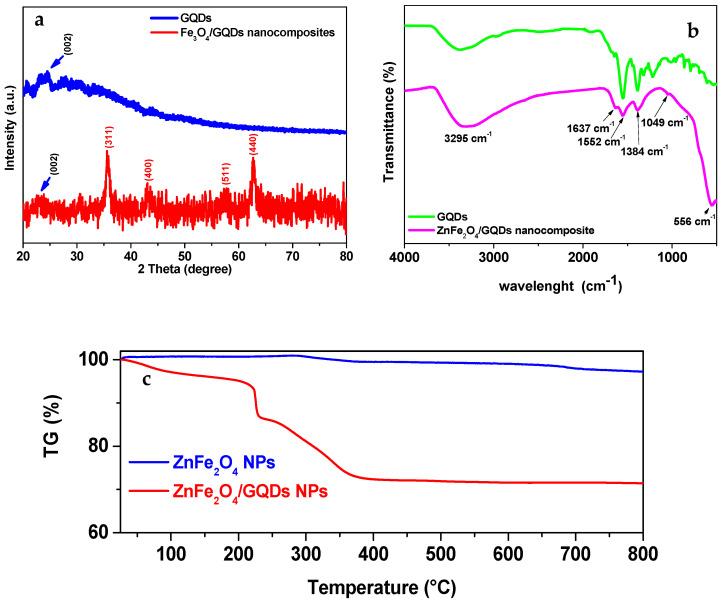
XRD patterns of GQDs and ZnFe_2_O_4_/GQDs NPs (**a**). FT-IR spectra of GQDs and ZnFe_2_O_4_/GQDs nanocomposite (**b**). TG profiles of ZnFe_2_O_4_ NPs alone (blue line) and ZnFe_2_O_4_/GQDs (red line) nanocomposite (**c**).

Figure 2a displays the X-ray diffraction spectra of the synthesized GQDs and ZnFe_2_O_4_/GQDs NPs. GQDs exhibit a faint broad (002) peak, attributed to the thin and irregular stacking of certain GQDs [55]. The XRD pattern of the ZnFe_2_O_4_/GQDs NPs shows diffraction peaks at 2θ =35.8°, 44.1°, 57.5°, and 63.1°, which can be assigned to the (311), (400), (511), and (440) planes of ZnFe_2_O_4_ NPs [56], respectively. This indicates that the ZnFe_2_O_4_ nanoparticles in the ZnFe_2_O_4_/GQDs NPs were phase-pure with a cubic spinel structure, in good agreement with the standard ZnFe_2_O_4_ (JCPDS card No. 01-077-0011). Moreover, the presence of GQDs does not affect the structure of ZnFe_2_O_4_ nanoparticles.

### 3.3. FT-IR Studies

Figure 2b illustrates the FT-IR spectra of GQDs and ZnFe_2_O_4_ NPs. The prominent broad band at 3365 cm^−1^ is attributed to the stretching vibration of -OH groups [57]. Additionally, peaks at 2971 cm^−1^ and 2917 cm^−1^ correspond to the asymmetric and symmetric C-H stretching vibrations, respectively. The band observed at 1558 cm^−1^ is associated with bending vibrations of the C=C group. Furthermore, intense bands at 1654 cm^−1^, 1395 cm^−1^, and 1223 cm^−1^ are assigned to the C=O, C-O (carboxyl), and C-O (alkoxy) functional groups, respectively [58].

In the FT-IR spectrum of ZnFe_2_O_4_/GQDs NPs, a distinct stretching vibration band at 556 cm^−1^ for M-O indicates the presence of the metal [59]. Moreover, after the formation of the nanocomposites, there is a shift in vibrational bands, indicating interaction between GQDs and magnetic nanoparticles. Particularly, the stretching vibration band of C=O groups shifts from 1654 cm^−1^ to 1637 cm^−1^, suggesting that carboxylic groups on the edges of GQDs have been converted to carboxylate groups [60]. This shift confirms the binding of GQDs to ZnFe_2_O_4_ NPs through M-O chemical bonds [61]. The BET-specific surface area (BET-SSA) of the ZnFe_2_O_4_/GQDs NPs, which can provide numerous adsorption sites for TMB, was determined to be 58 m^2^/g. The thermal degradation profiles of bare ZnFe_2_O_4_ and ZnFe_2_O_4_@GQDs are shown in Figure 2c. As indicated by the TGA curves, ZnFe_2_O_4_@GQDs exhibits a significant weight loss of approximately 30% starting above 200 °C, which can be attributed to the thermal decomposition of the graphene quantum dots coating the nanoparticles.

### 3.4. Morphological Characterization

**Figure 3:**
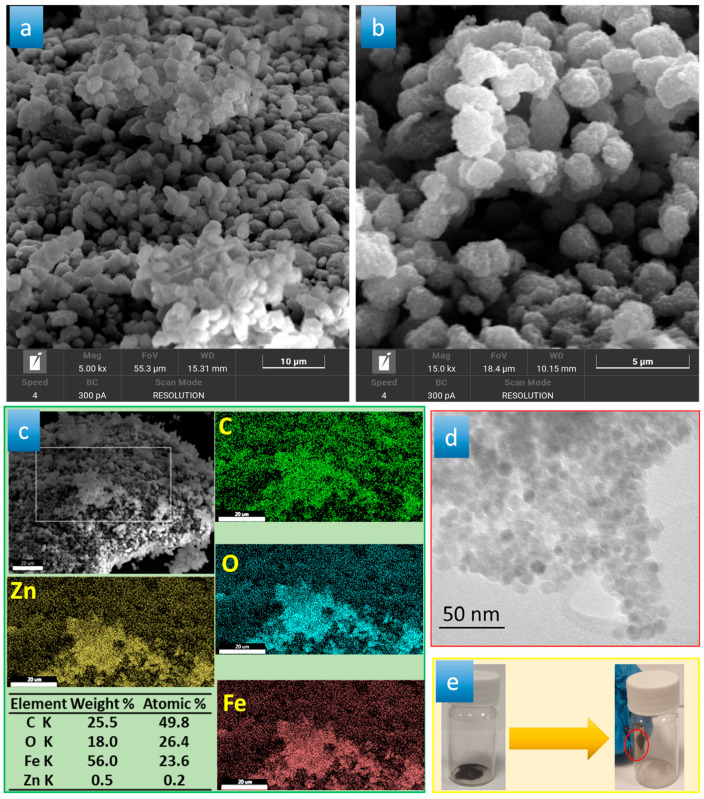
SEM images of ZnFe_2_O_4_/GQDs NPs (**a**,**b**); elemental maps of different elements and quantification by energy-dispersive X-ray spectroscopy analysis (**c**); TEM image of ZnFe_2_O_4_/GQDs NPs (**d**); photographs showing the magnetic behavior of ZnFe_2_O_4_/GQDs NPs without and with the application of a magnetic field (**e**).

The particle size distributions and morphologies of both GQDs alone and ZnFe_2_O_4_/GQDs NPs are examined through microscopy observations (Figure 3 and Figure S1). The morphological analysis of GQDs shows a good nanostructure dispersion and a quasi-spherical morphology with an average size of 3.2 ± 1.63 nm. Figure 3a,b show SEM images of NP aggregates with quasi-spherical shapes and an average size of a few hundred nanometers. The presence of carbon (C), oxygen (O), zinc (Zn), and iron (Fe) elements in the magnetic nanocomposites is clearly demonstrated by the elemental mapping images shown in Figure 3c, suggesting uniform GQDs distribution and successful nucleation and growth of ZnFe_2_O_4_ NPs. Figure 3d further displays a TEM image of the ZnFe_2_O_4_/GQDs NPs, showing the resulting nanocomposites composed of nanoparticles approximately 10 nm in diameter; in some cases, a light halo results, which is visible likely due to GQDs at the nanoparticles periphery. Figure 3e shows the magnetic nanocomposite powder. In particular, the particles can be dragged by a permanent magnet, clearly demonstrating the magnetic behavior of the ZnFe_2_O_4_/GQDs NPs.

### 3.5. Peroxidase-like Activity of ZnFe_2_O_4_/GQDs Nanoparticles

**Figure 4:**
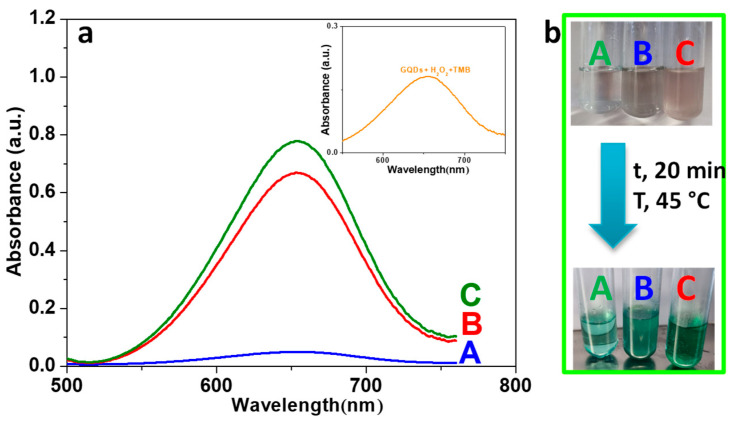
UV–vis absorption spectra of TMB-H_2_O_2_ systems: TMB + H_2_O_2_ (A); ZnFe_2_O_4_ + TMB + H_2_O_2_ (B); ZnFe_2_O_4_/GQDs + TMB + H_2_O_2_ (C) (**a**); UV–vis absorption spectra of GQDs + TMB-H_2_O_2_ (see inset (**a**)); and typical photographs, in HAc-NaAc buffer at pH 4, before and after incubation at 45 °C for 20 min (**b**). Conditions: TMB [10 mM], H_2_O_2_ [5 mM]; pH, 4.0; catalyst, 1 mg/mL; temperature, 45 °C; time, 20 min.

To investigate the peroxidase-like capabilities of the synthesized ZnFe_2_O_4_/GQDs NPs and the role of GQDs, catalytic oxidations of the TMB substrate were conducted using both ZnFe_2_O_4_ NPs alone and ZnFe_2_O_4_/GQDs NPs in the presence of H_2_O_2_, as illustrated in Figure 4a. The results demonstrated that ZnFe_2_O_4_ NPs, GQDs, and ZnFe_2_O_4_/GQDs NPs were capable of facilitating the oxidation of TMB to ox-TMB under identical reaction conditions, with maximum absorbance observed at 652 nm. This behavior resembles the peroxidase-like activity of enzymes like HRP [62]. Specifically, the absorbance at 652 nm of the ZnFe_2_O_4_/GQDs NPs + TMB + H_2_O_2_ system (sample C) was higher compared to the other tested systems (Figure 4a), indicating enhanced peroxidase-like activity resulting from the combined contribution of ZnFe_2_O_4_ and GQDs, even when compared to GQDs alone, which exhibited moderate peroxidase-like activity in the presence of H_2_O_2_ and TMB (see inset in Figure 4a). Moreover, the notable color change observed in the ZnFe_2_O_4_/GQDs NPs + TMB + H_2_O_2_ (A_652_ = 0.784 ± 0.14) system was greater than that of the ZnFe_2_O_4_ NPs + TMB + H_2_O_2_ (sample B) (A_652_ = 0.673 ± 0.09) system under identical conditions (see Figure 4b), indicating the strong catalytic activity of ZnFe_2_O_4_/GQDs NPs. These findings suggest that the synthesized ZnFe_2_O_4_/GQDs NPs exhibit good peroxidase-like activity, attributed to the intact aromatic structure and the rich carboxylic groups on the periphery of GQDs [63,64], enhancing catalytic efficiency through a synergistic mechanism as further confirmed by control experiments with physical mixtures of the two components, which showed lower activity, likely due to competitive interactions between individual species (see Figure S3).

### 3.6. Experimental Conditions Optimization

Similar to natural enzymes and other reported nanomaterial-based peroxidase mimetics, certain conditions, such as time, pH, temperature, and catalyst concentration, might affect the catalytic activity. In particular, their effect on the catalytic activity of ZnFe_2_O_4_/GQDs NPs was studied. The catalytic activity of ZnFe_2_O_4_/GQDs NPs was measured in the pH range from 2 to 10, with temperatures from 30 °C to 60 °C, and catalyst concentrations from 0.2 mg/mL to 2 mg/mL.

**Figure 5:**
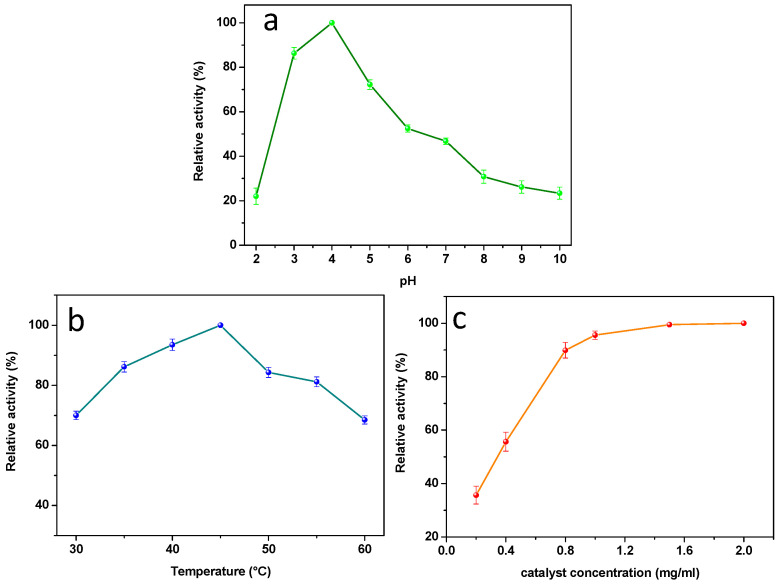
Dependency of the ZnFe_2_O_4_@GQDs NPs peroxidase-like activity on pH (**a**), temperature (**b**), and the ZnFe_2_O_4_@GQDs NPs concentration (**c**). Conditions: TMB [10 mM]; H_2_O_2_ [5 mM]; time, 20 min. The absorbance was read at the maximum absorbance of 652 nm. The maximum point in each curve was set as 100%. The error bars represent the standard deviation of three measurements.

As shown in Figure 5, the relative activity of ZnFe_2_O_4_/GQDs NPs increased when the pH was increased from 2.0 to 4.0 and decreased for pH > 4.0 (Figure 5a). Therefore, the maximum relative activity was at pH 4. This result is in agreement with reported experiments that low acidic conditions are ideal for oxidation of the TMB-H_2_O_2_ system [65]. The curve shows improved stability, i.e., in a wider pH range, compared to the typical behavior of enzymes and other mimics [65,66]. This broadening of the pH working range is likely due to the intrinsically acidic surface behavior induced by the functional groups of GQDs, which provide appropriate microenvironments even in a neutral buffer for the catalytic reaction [67]. This is a particularly relevant aspect, considering that the GOx enzymatic reaction generally occurs under neutral pH conditions [68,69,70]. Like natural enzymes, the catalytic activity of the ZnFe_2_O_4_/GQDs NPs is also dependent on reaction temperature. In particular, the relative activity of ZnFe_2_O_4_/GQDs nanoparticles increased as the temperature rose from 30 °C to 45 °C, then decreased at higher temperatures up to 60 °C (Figure 5b), indicating that the optimal temperature is 45 °C. This behavior is mainly because both TMB and H_2_O_2_ become unstable at elevated temperatures [71]. Specifically, H_2_O_2_ tends to decompose more rapidly, which negatively affects the oxidation of TMB molecules [72], thereby reducing the overall catalytic efficiency. In addition, increased temperature can lead to changes in the surface structure or electronic properties of the nanoparticles, potentially altering the accessibility or reactivity of active sites. Thermal fluctuations may also affect the adsorption–desorption equilibrium between the substrate and the catalyst surface, leading to a decrease in reaction rate at higher temperatures [73]. Nonetheless, ZnFe_2_O_4_/GQDs nanoparticles still exhibit considerable catalytic activity across the 30–60 °C range, highlighting their structural robustness compared to natural enzymes and their potential applicability in harsh environments. As shown in Figure 5c, as the amount of ZnFe_2_O_4_/GQDs NPs rose from 0.2 to 0.8 mg/mL, the relative activity gradually increased, favoring the continuous formation of radical active groups. At higher concentrations, the increase of ZnFe_2_O_4_/GQDs NP peroxide mimetic enzyme amount determines further light rising. However, the relative activity remained relatively unchanged from 1 mg/mL to 2 mg/mL, likely due to the magnetic nature of the ZnFe_2_O_4_/GQDs NPs mimetic enzyme. Indeed, the larger amount of mimic enzymes favors magnetic nanoparticle aggregation, reducing the available specific surface area of the nanoparticles participating in the catalytic reaction [72]. Thus, the optimal amount of ZnFe_2_O_4_/GQDs NPs mimic enzyme was 1 mg/mL. As a further comparison, the optimal conditions for ZnFe_2_O_4_ NPs alone were investigated. As shown in Figure S2, ZnFe_2_O_4_ NP nanoparticles exhibited the highest activity when the pH was set as 4.0, which was like that of ZnFe_2_O_4_/GQDs NPs. However, the catalytic activity of ZnFe_2_O_4_ NPs dramatically declined in alkali conditions and at temperatures higher than 40 °C. Therefore, the robustness of ZnFe_2_O_4_/GQDs NPs, making them potentially applicable under harsh conditions, is strongly improved by the protective layer associated with the presence of the GQDs.

### 3.7. Steady-State Kinetic Assay of ZnFe_2_O_4_/GQDs Nanoparticles

**Figure 6:**
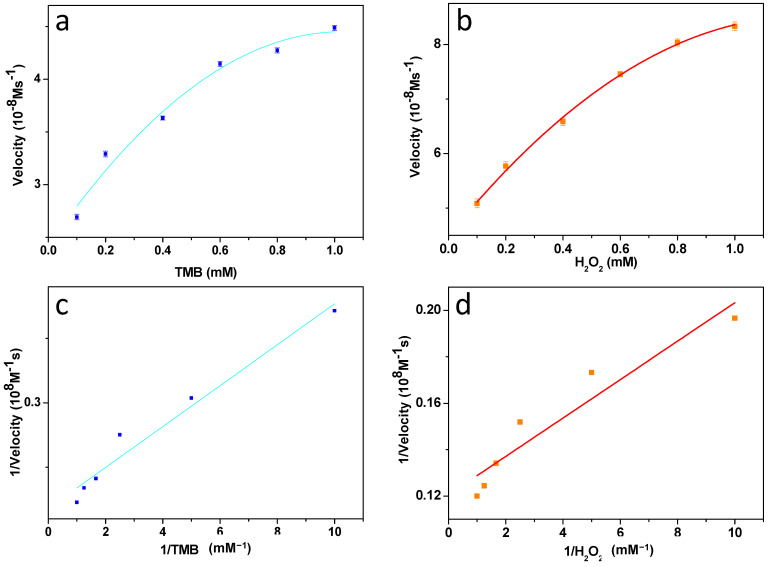
Steady-state kinetic assays of ZnFe_2_O_4_@GQDs by using the Michaelis–Menten model at a constant concentration of H_2_O_2_ [5.0 mM], while TMB concentration was varied from 0.1 to 1.0 mM (**a**), and at a constant concentration of TMB [0.8 mM], while H_2_O_2_ concentration was varied from 0.1 to 1.0 mM (**b**); double-reciprocal plots of initial velocity versus TMB concentration (**c**); and double-reciprocal plots of initial velocity versus H_2_O_2_ concentration (**d**). Conditions: pH 4.0; catalyst 80 µg/mL.

To explore the kinetic mechanism of the nanoparticles’ peroxidase-like activity, the apparent steady-state kinetic parameters of the peroxidase-like color reaction were determined by varying the concentrations of TMB and H_2_O_2_ in the system. Kinetic experiments were conducted using 80 μg/mL ZnFe_2_O_4_@GQDs in a reaction volume of 3.0 mL in HAc-NaAc buffer solution (pH 4.0), with 0.8 mM TMB as the substrate and 5 mM H_2_O_2_ unless otherwise specified. The Km and Vmax values were derived using the Lineaweaver–Burk double reciprocal plot (Figure 6). The obtained Km value for the easily recoverable magnetic ZnFe_2_O_4_@GQDs NPs (see Table 1 and references [19,22,23,44,45,66,74,75,76,77,78,79,80,81]) underscores their strong affinity toward both H_2_O_2_ and TMB as substrates, facilitated by the synergistic functions of GQDs and ZnFe_2_O_4_. This characteristic allows for practical applications in the detection of H_2_O_2_ concentrations [82].

### 3.8. Catalytic Mechanism

**Scheme 2:**
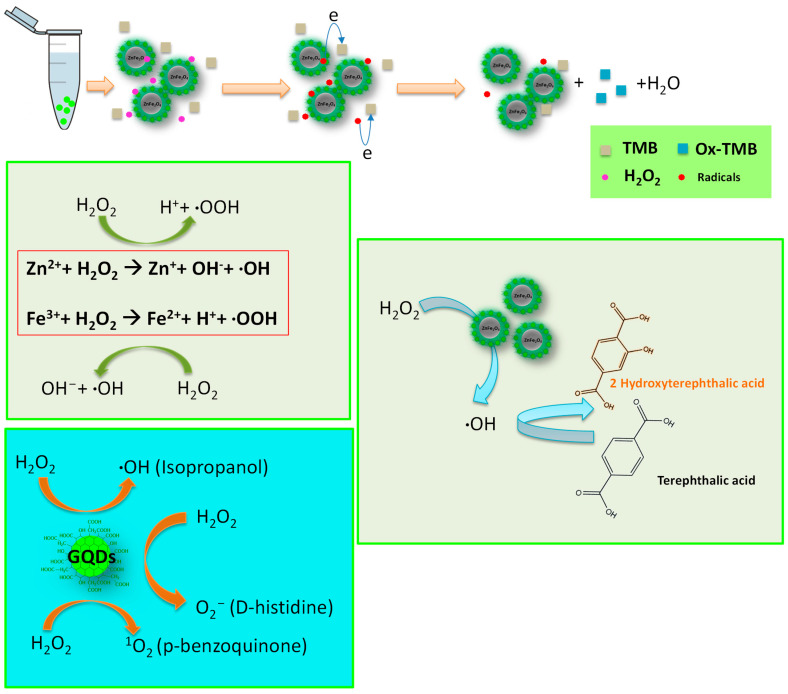
The catalytic mechanism for the ZnFe_2_O_4_/GQDs NPs-H_2_O_2_-TMB system and depictions of ZnFe_2_O_4_/GQDs NPs-H_2_O_2_ produced ROS.

The catalytic mechanism of ZnFe_2_O_4_/GQDs NPs in the presence of H_2_O_2_ and TMB can be described by a ping-pong reaction mechanism, involving the generation of ROS and electron transfer processes, as depicted in the top of Scheme 2 [83].

GQDs act as protective shells around ZnFe_2_O_4_ nanoparticles, preventing damage and aggregation. To understand the role of GQDs in the peroxidase mimic activity of ZnFe_2_O_4_@GQDs NPs, experiments were conducted with GQDs alone (Figure S4). Both H_2_O_2_ and GQDs individually show minimal ability to oxidize TMB, reflecting their low intrinsic catalytic efficiency when used separately (Figure S4a), thereby highlighting the essential role of the interaction between GQDs, H_2_O_2_, and TMB in the catalytic reaction. The absorption spectra revealed a bathochromic shift in GQDs after adding H_2_O_2_ (Figure S4b), similar to the behavior observed with graphene oxide materials [78]. This shift indicates electron transfer from the top of the valence band of the nanocarbon matrix to the lowest unoccupied molecular orbital of H_2_O_2_. TMB binds to the surface of GQDs and donates lone-pair electrons from amino groups to GQDs, enhancing electron density and mobility. This n-type doping of GQDs increases the Fermi level and electrochemical potential, facilitating electron transfer from GQDs to H_2_O_2_ [84,85,86]. Nitrogen enrichment further enhances the concentration of catalytically active sites and minimizes steric hindrance for redox species binding.

Additionally, Zn^2+^ and Fe^3+^ ions can react with H_2_O_2_ to produce intermediate hydroxyl (·OH) radicals, which oxidize TMB to its blue-colored form. Overall, these results demonstrate that ZnFe_2_O_4_@GQDs NPs possess intrinsic peroxidase-like activity, with catalytic performance influenced by pH, temperature, and H_2_O_2_ concentration akin to horseradish peroxidase. In the presence of H_2_O_2_ and TMB, ZnFe_2_O_4_@GQDs NPs induce a blue color reaction, outperforming ZnFe_2_O_4_ NPs due to their higher surface-to-volume ratios and affinity to organic substances via π-π and hydrophobic interactions.

Furthermore, to confirm that the peroxidase-like activity of ZnFe_2_O_4_/GQDs NPs stems from the generation of highly reactive ·OH radicals, their presence was monitored using terephthalic acid, which forms 2-hydroxy terephthalic acid in the presence of OH radicals [60], as depicted in Scheme 2. Figure S5a shows a significant fluorescence peak at 428 nm after 5 min, indicating the formation of ·OH radicals and subsequent production of fluorescent 2-hydroxy terephthalic acid.

Moreover, the peroxidase-mimicking activity of ZnFe_2_O_4_/GQDs NPs may involve the generation of other ROS, such as singlet oxygen (^1^O_2_) and superoxide radicals (O_2_•−). To further investigate this mechanism, specific ROS scavengers, including isopropanol, P-benzoquinone, and D-histidine, were employed to track ·OH, O_2_•−, and ^1^O_2_, respectively (Scheme 2). Figure S5b shows a noticeable decrease in absorbance at 652 nm when ROS scavengers were added to both GQDs + TMB + H_2_O_2_ and ZnFe_2_O_4_@GQDs NPs + TMB + H_2_O_2_ systems, indicating the presence of generated O_2_•−, ^1^O_2_, and ·OH radicals. These findings underscore the role of Fe_2_O_4_ in catalyzing these reactions and validate the diverse ROS generation capabilities of ZnFe_2_O_4_/GQDs NPs.

**Table 1:** Comparison of the apparent Michaelis–Menten constant (Km) and maximum reaction rate (Vm) of different enzyme mimics.

Catalyst	Substrate	*V_max_* (×10^−8^ M/s)	*K_m_* (mM)	References
Fe_3_O_4_@NH_2_-MIL-101(Fe)	TMB	10.7	0.246	[77]
Fe_3_O_4_@NH_2_-MIL-101(Fe)	H_2_O_2_	3.65	0.105	[77]
HRP	TMB	10.0	0.434	[19]
HRP	H_2_O_2_	8.71	3.70	[19]
Fe_3_O_4_	TMB	3.44	0.0980	[19]
Fe_3_O_4_	H_2_O_2_	9.78	154	[19]
GO-Fe_3_O_4_	TMB	13.08	0.430	[78]
GO-Fe_3_O_4_	H_2_O_2_	5.31	0.710	[78]
CuNPs@C	TMB	12.1	1.65	[79]
CuNPs@C	H_2_O_2_	5.30	1.89	[79]
oS_2_- Pt_74_Ag_26_	TMB	7.29	25.7	[86]
MoS_2_- Pt_74_Ag_26_	H_2_O_2_	3.22	0.386	[80]
CDs@Fe_3_O_4_	TMB	66.67	0.17	[65]
CDs@Fe_3_O_4_	H_2_O_2_	2.21	3.16	[65]
FeSe film	TMB	8.90	0.04	[74]
FeSe film	H_2_O_2_	15.40	13.20	[74]
Fe_3_O_4_@CeO_2_ NCs	TMB	0.64	0.15	[81]
Fe_3_O_4_@CeO_2_ NCs	H_2_O_2_	12.5	1.13	[81]
ZnFe_2_O_4_	TMB	13.31	0.85	[23]
ZnFe_2_O_4_	H_2_O_2_	7.74	1.66	[22]
5-Fe-MSN	TMB	0.331	0.122	[76]
5-Fe-MSN	H_2_O_2_	0.3267	6.67	[76]
N-GQDs	TMB	0.38	11.90	[45]
N-GQDs	H_2_O_2_	0.14	0.1	[45]
o-GQDs	TMB	8.389	0.1858	[44]
o-GQDs	H_2_O_2_	7.75	0.1363	[44]
ZnFe_2_O_4_/GQDs NPs	TMB	4.58	0.072	This work
ZnFe_2_O_4_/GQDs NPs	H_2_O_2_	8.29	0.068	This work

### 3.9. Detection of H_2_O_2_ and Glucose

**Figure 7:**
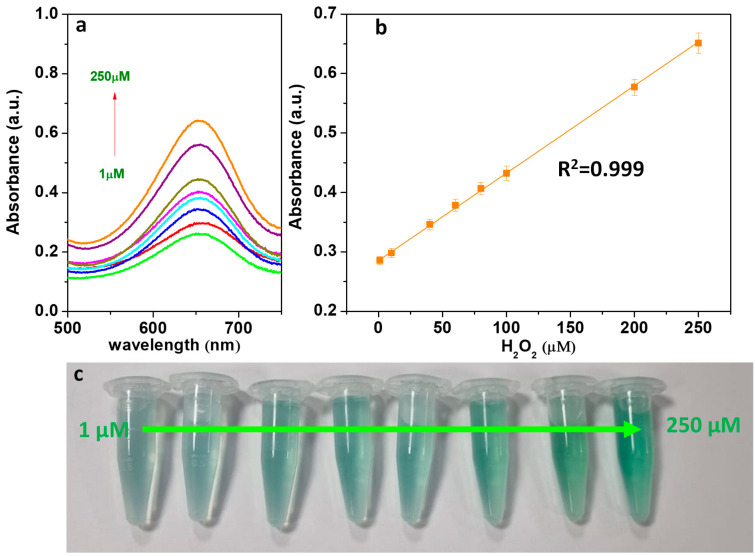
(**a**) The UV–vis absorption spectra of the solution after the reaction by ZnFe_2_O_4_@GQDs NPs/TMB system at various concentrations of H_2_O_2_. (**b**) Absorbance monitoring at 652 nm as a function of H_2_O_2_ concentration. (**c**) The corresponding photographs of the reaction solutions at different concentrations of H_2_O_2_, ranging from 1 to 250 μM. Conditions: TMB [10 mM]; H_2_O_2_ [1–250 μM]; pH, 4.0; catalyst, 1 mg/mL; temperature, 45 °C; time, 20 min.

**Figure 8:**
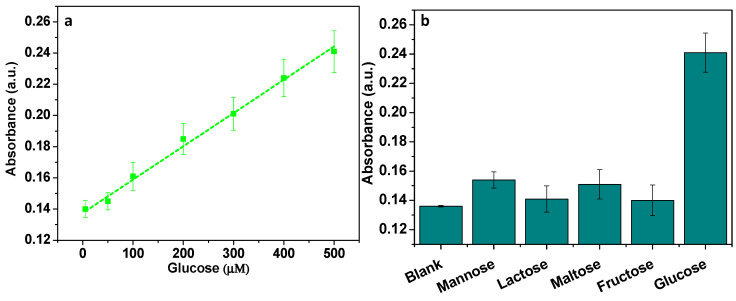
(**a**). Calibration curve of absorbance at 652 nm vs. glucose concentration. Conditions: TMB [10 mM], glucose [5–500 μM]; pH, 4.0; catalyst, 1 mg/mL; GOx, 1 mg/mL; temperature, 45 °C; time, 20 min (**b**). Determination of the specificity of glucose detection for the TMB + ZnFe_2_O_4_@GQDs + GOx system in the absence (blank) and presence of glucose (0.5 mM), mannose (5 mM), maltose (5 mM), lactose (5 mM), and fructose (5 mM)

Under the optimized conditions outlined above, the sensitivity of ZnFe_2_O_4_@GQDs NPs for detecting H_2_O_2_ and glucose was assessed. Figure 7a illustrates the absorbance curves at 652 nm corresponding to different H_2_O_2_ concentrations measured over 20 min. The plot in Figure 7b shows the linear relationship between absorbance at 652 nm and H_2_O_2_ concentration when using ZnFe_2_O_4_@GQDs NPs as a peroxidase-like catalyst, demonstrating a good linear response from 1 µM to 0.25 mM of H_2_O_2_ concentration. This linear relationship is a quantitative method for H_2_O_2_ detection, with a detection limit (LOD) of 3 µM and a naked-eye detection limit of 10 μM. Thus, ZnFe_2_O_4_@GQDs exhibit robust peroxidase-like activity at low concentrations, making them highly effective as colorimetric reagents. This system was also utilized for glucose detection using glucose oxidase (GOx). In the presence of GOx, glucose is converted to gluconic acid with the concurrent generation of H_2_O_2_, which interacts with the substrate and NPs, leading to a color change (see Scheme 1). Figure 8a shows the relationship between absorbance intensity and glucose concentration in the solution, demonstrating a proportional relationship over the range of 5–500 μM (R^2^ = 0.99), with a detection limit of 7 μM. Comparatively, the performance of other similar peroxidase mimics for glucose detection is summarized in Table 2 [53,87,88,89,90]. ZnFe_2_O_4_@GQDs NPs exhibit a good glucose detection range compared to other nano-enzymes.

To assess the specificity of the method, control experiments were conducted using several carbohydrates (lactose, sucrose, fructose, maltose, and mannose) instead of glucose. Figure 8b shows that these carbohydrates, even at ten times higher than glucose, did not yield significant absorbance intensity, whereas glucose produced a distinctly higher absorbance. This confirms the high selectivity of the developed method for glucose detection, attributed to the high specificity of GOx towards glucose in catalytic oxidation.

### 3.10. Analysis of Glucose in Real Blood Samples

Glucose detection in human blood serum is crucial for the diagnosis of diabetes. Therefore, the applicability of the colorimetric method based on ZnFe_2_O_4_@GQDs NPs was investigated for determining glucose concentration in three human serum samples (Table 3). The glucose concentrations were determined from the calibration curve, and these results are consistent with the values estimated by standard clinical laboratory methods. Typically, blood glucose concentrations range from approximately 3 to 8 mM in healthy individuals and from 9 to 40 mM in diabetic patients [91]. Hence, the colorimetric method shows significant potential for clinical glucose analysis, underscoring its practical utility in real-world applications.

### 3.11. Reusability of ZnFe_2_O_4_@GQDs Nanoparticles

**Figure 9:**
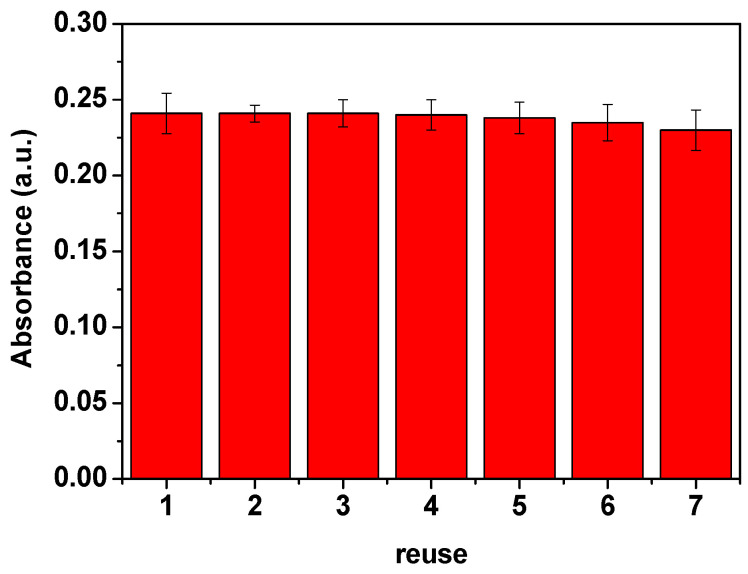
Reusability of ZnFe_2_O_4_@GQDs after repeated cycles of glucose detection using identical reaction conditions. Conditions: TMB [10 mM]; glucose [0–500 μM]; pH, 4.0; catalyst, 1 mg/mL; GOx, 1 mg/mL; temperature, 45 °C; time, 20 min.

The reusability of the prepared nanocomposite was also assessed. As shown in Figure 9, there was no significant decrease in absorbance when the magnetic composites were utilized for six consecutive cycles. The small decrease in the absorbance observed is likely due to slight sample loss during the cycles. Furthermore, ZnFe_2_O_4_@GQDs nanoparticles exhibited higher stability and recyclability than ZnFe_2_O_4_ nanoparticles; e.g., after six reuse cycles of ZnFe_2_O_4_ NPs, a reduction of approximately 10% in absorbance was observed, highlighting the role of GQDs in stabilizing the catalyst and improving recovery.

Finally, to eliminate the possibility that enzymatic activity resulted from the leaching of free Zn^2+^ ions or Fe^3+^ ions into the buffer, ZnFe_2_O_4_@GQDs nanoparticles were incubated in the HAc-NaAc buffer (pH 4.0) for 24 h. The supernatant was collected by removing ZnFe_2_O_4_@GQDs NPs through magnetic separation. Subsequently, the same experimental procedure (as described in the H_2_O_2_ detection section, Section 2.5) was followed by taking 500 µL of the centrifuged solution as the catalyst. Figure S6 shows the catalytic activity of H_2_O_2_ + TMB alone in comparison with H_2_O_2_ + TMB + “potentially leached ions”, indicating the absence of leached ions in the solution that could contribute to catalytic activity in the presence of H_2_O_2_ and TMB (see Figure S6). This supports the stabilizing role of GQDs, which not only improves the durability of the catalyst but also enhances its recyclability.

## 4. Conclusions

ZnFe_2_O_4_ nanoparticles, nucleated in situ and coated with GQDs, were synthesized for H_2_O_2_ and glucose detection. SEM-EDX analysis confirmed the presence of carbon, oxygen, zinc, and iron elements, indicating a uniform distribution of GQDs and successful ZnFe_2_O_4_ growth. TEM images revealed quasi-spherical nanocomposites with well-defined morphology. These materials were tested on simulated and real serum samples, exhibiting linear detection ranges of 1–250 µM for H_2_O_2_ and 5–500 µM for glucose. Their robust performance across varying pH conditions was attributed to the acidic surface behavior of GQDs, supporting catalytic activity even in neutral buffer environments.

Lineweaver–Burk analysis yielded favorable Km and Vmax values, indicating strong substrate affinity and high catalytic efficiency. Extremely low detection limits were achieved, owing to the synergistic interaction between ZnFe_2_O_4_ and GQDs. Reusability tests showed no significant loss in performance after six cycles. The presence of GQDs improved the composite’s stability, limiting the decrease in absorbance to only 10%, compared to ZnFe_2_O_4_ alone. These results underline the nanocomposite’s stability, recyclability, and practical potential for real-world scenarios.

## Figures and Tables

**Table 2:** Comparison with other state-of-the-art catalysts for glucose detection, from the linear range and detection limit.

State-of-the-Art Catalysts	Detection Limit (μM)	Linear Range (μM)	References
CTAB@MgFe_2_O_4_	5	10–1000	[87]
PTB-GOx	22.2	75–7500	[88]
Fe_3_O_4_@COFs	6.0	50–900	[90]
C/CdTe	223	0–13,000	[89]
Co_3_O_4_	5	10–1000	[53]
ZnFe_2_O_4_/GQDs NPs	7.0	5–500	This work

**Table 3:** Comparison of the glucose contents in serum samples by the current colorimetric method and provided by the volunteers.

Sample	Current Colorimetric Method (mM ± SD, *n* = 3)	Standard Pathological Laboratory Method (mM)
Serum 1	4.65 ± 0.15	4.72
Serum 2	4.57 ± 0.21	4.61
Serum 3	5.83 ± 0.08	5.89

## Data Availability

The authors confirm that the data supporting the findings of this study are available within the article. Raw data supporting the findings of this study are available from the corresponding author on request.

## Supplementary Materials

The following supporting information can be downloaded at: https://www.mdpi.com/article/10.3390/mi16050520/s1. Figure S1: TEM images of GQDs. Figure S2: ZnFe2O4 NP peroxidase-like activity dependence by pH (a) and temperature (b). Conditions: TMB [10 mM]; H2O2, [5 mM]; time, 20 min; catalyst concentration [1 mg/ml]. The absorbance was read at the maximum absorbance of 652 nm. The maximum point in each curve was set as 100. The error bars represent the standard deviation of three measurements. Figure S3. UV–vis absorption spectra of ZnFe2O4/GQDs NPs+TMB+H2O2 and ZnFe2O4 NPs+GQDs+TMB+H2O2 systems. Conditions: TMB [10 mM], H2O2 [5 mM]; pH, 4.0; catalyst, 1 mg/mL; temperature, 45 °C;; time, 20 min. Figure S4. UV–vis absorption spectra of H2O2+TMB, GQDs+TMB, and GQDs+H2O2+TMB at pH 4 (a). Conditions: TMB, [10 mM]; H2O2, [5 mM]; pH, 4.0; catalyst, 1 mg/ml; temperature, 45 °C; time, 20 min. UV–vis absorption spectra of GQDs and GQDs+H2O2 at pH 4 after incubation at 45 °C for 20 min (b). Figure S5. Terephthalic acid-based test for hydroxyl radicals monitoring of fluorescence (PL = photoluminescence) signal after 5 min in the presence of ZnFe2O4/GQDs NPs (a). Determination of reactive oxygen species in GQDs and ZnFe2O4/GQDs NPs with D-histidine, isopropyl alcohol, and p-benzoquinone as ROS scavengers (b). Figure S6. UV–vis absorption spectra of TMB+H2O2, leaching ions of ZnFe2O4@GQDs NPs and ZnFe2O4 NPs+TMB+H2O2. Conditions: TMB, [10 mM]; H2O2, [5 mM]; pH, 4.0; temperature, 45 °C;; time, 20 min.
