# Supplementary material for: ZnFe2O4/GQDs Nanoparticles as Peroxidase Mimics for Sensitive and Selective Colorimetric Detection of Glucose in Real Samples
Source: Micromachines (Basel). 2025 Apr 28;16(5):520. doi: 10.3390/mi16050520 (PMC12113822; doi:10.3390/mi16050520)
Supplement: Supplementary file 1 [file micromachines-16-00520-s001.zip › micromachines-3555488-supplementary.pdf]

Supplementary materials

$ZnFe_2O_4$/GQDs nanoparticles as peroxidase mimics for sensitive and selective colorimetric detection of glucose in real samples

Claudia Cirillo[1,2†*], Mariagrazia Iuliano[1,2†], Maria Sarno[1,2]

[1]*Department of Physics "E.R. Caianiello" University of Salerno, Via Giovanni Paolo II, 132 - 84084 Fisciano (SA), Italy.*

[2]*NanoMates, Research Centre for Nanomaterials and Nanotechnology at the University of Salerno, University of Salerno, Via Giovanni Paolo II, 132 - 84084 Fisciano (SA), Italy.*

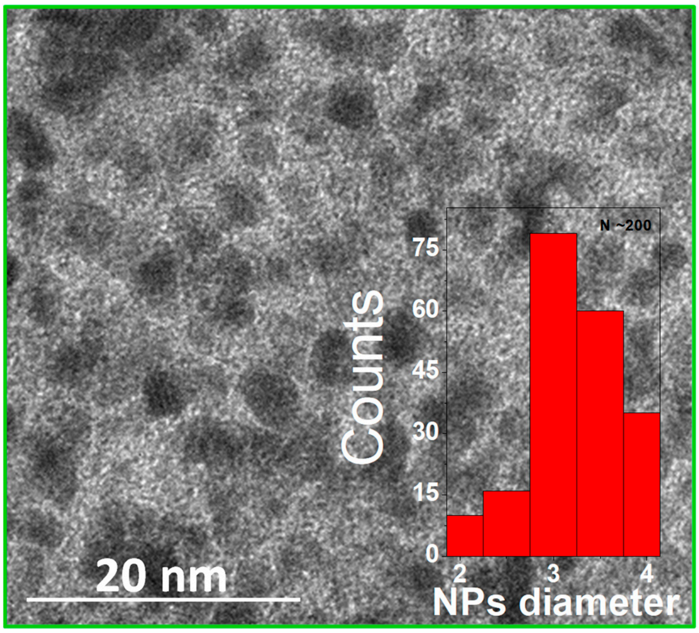

Figure S1.TEM images of GQDs.

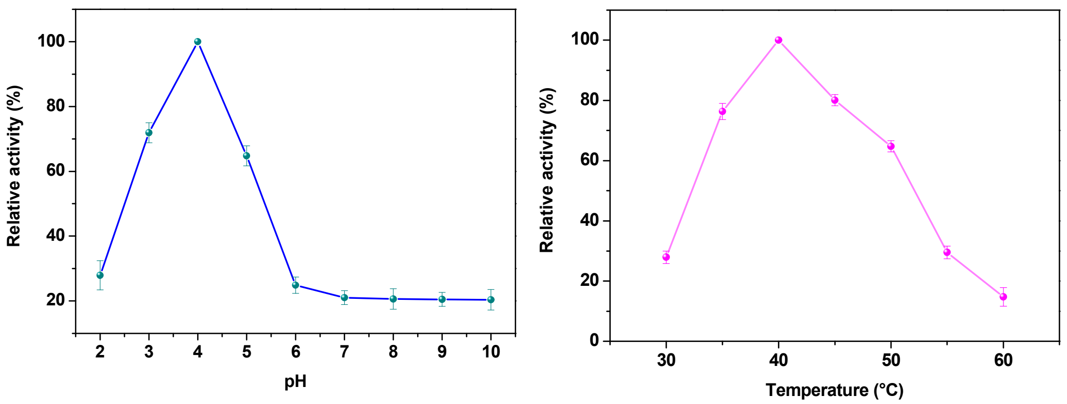

Figure S2. $ZnFe_2O_4$ NP peroxidase-like activity dependence by pH (a) and temperature (b). Conditions: TMB [10 mM]; H_2O_2, [5 mM]; time, 20 min; catalyst concentration [1 mg/ml]. The absorbance was read at the maximum absorbance of 652 nm. The maximum point in each curve was set as 100. The error bars represent the standard deviation of three measurements.

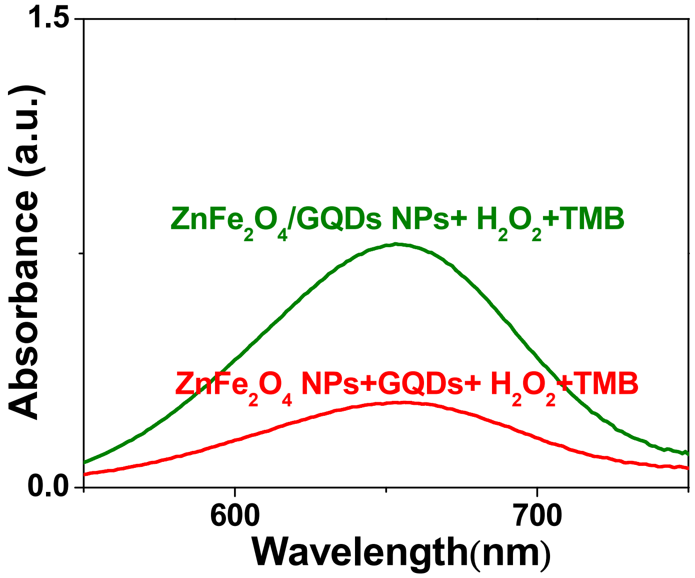

Figure S3. UV–vis absorption spectra of $ZnFe_2O_4$/GQDs NPs+TMB+H_2O_2 and $ZnFe_2O_4$ NPs+GQDs+TMB+H_2O_2 systems. Conditions: TMB [10 mM], H_2O_2 [5 mM]; pH, 4.0; catalyst, 1 mg/mL; temperature, 45°C; time, 20 min.

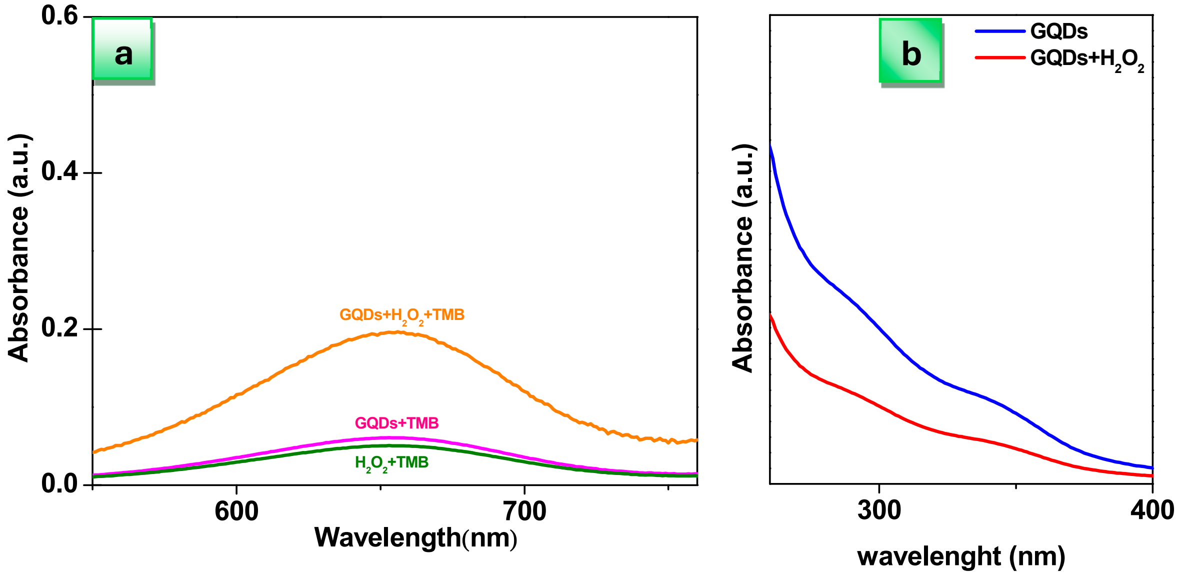

Figure S4. UV–vis absorption spectra of H_2O_2+TMB, GQDs+TMB, and GQDs+H_2O_2+TMB at pH 4 (a). Conditions: TMB, [10 mM]; H_2O_2, [5 mM]; pH, 4.0; catalyst, 1 mg/ml; temperature, 45°C; time, 20 min. UV–vis absorption spectra of GQDs and GQDs+H_2O_2 at pH 4 after incubation at 45 °C for 20 min (b).

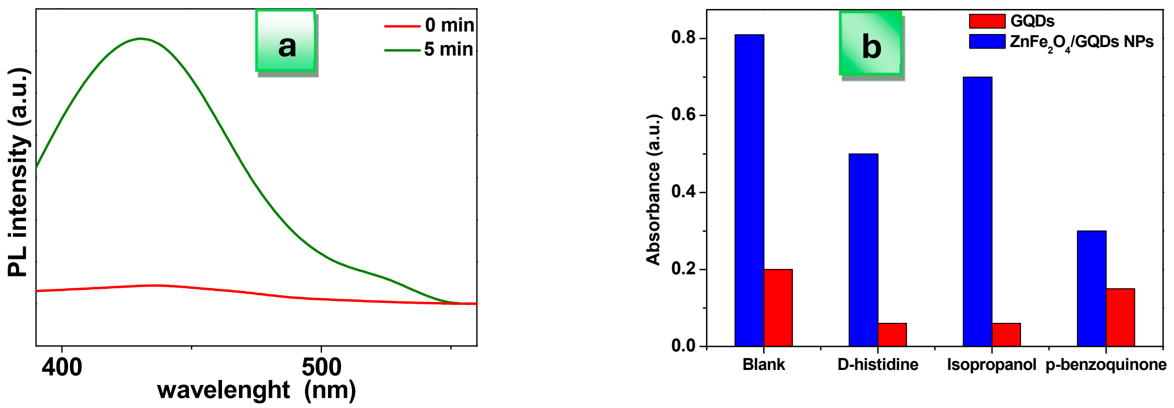

Figure S5. Terephthalic acid-based test for hydroxyl radicals monitoring of fluorescence (PL = photoluminescence) signal after 5 min in the presence of $ZnFe_2O_4$/GQDs NPs (a). Determination of reactive oxygen species in GQDs and $ZnFe_2O_4$/GQDs NPs with D-histidine, isopropyl alcohol, and p-benzoquinone as ROS scavengers (b).

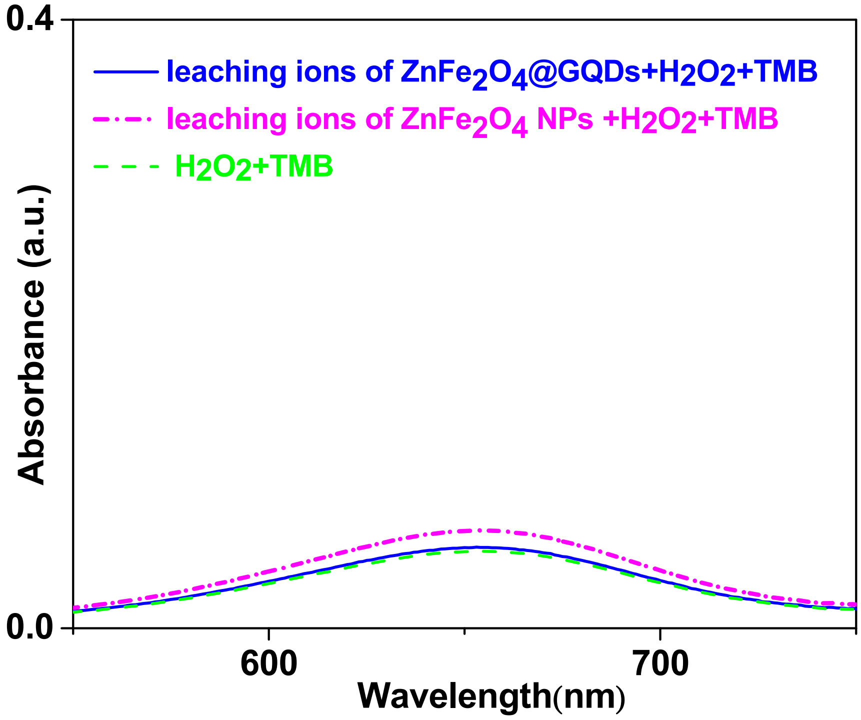

Figure S6. UV–vis absorption spectra of TMB+H_2O_2, leaching ions of $ZnFe_2O_4$@GQDs NPs and $ZnFe_2O_4$ NPs+TMB+H_2O_2. Conditions: TMB, [10 mM]; H_2O_2, [5 mM]; pH, 4.0; temperature, 45°C; time, 20 min.
